# Investigating the impact of human blood metabolites on the Sepsis development and progression: a study utilizing two-sample Mendelian randomization

**DOI:** 10.3389/fmed.2023.1310391

**Published:** 2023-12-08

**Authors:** Zhongqi Zhang, Yu Yin, Tingzhen Chen, Jinjin You, Wenhui Zhang, Yifan Zhao, Yankang Ren, Han Wang, Xiangding Chen, Xiangrong Zuo

**Affiliations:** ^1^Department of Critical Care Medicine, The First Affiliated Hospital of Nanjing Medical University, Nanjing, China; ^2^Department of Urology, The First Affiliated Hospital of Nanjing Medical University, Nanjing, China

**Keywords:** sepsis, metabolites, two-sample mendelian randomization, causal inference, mortality

## Abstract

**Background:**

Existing data suggests a potential link between human blood metabolites and sepsis, yet the precise cause-and-effect relationship remains elusive. By using a two-sample Mendelian randomization (MR) analysis, this study aims to establish a causal link between human blood metabolites and sepsis.

**Methods:**

A two-sample MR analysis was employed to investigate the relationship between blood metabolites and sepsis. To assess the causal connection between sepsis and human blood metabolites, five different MR methods were employed, A variety of sensitivity analyses were conducted, including Cochrane’s Q test, MR-Egger intercept test, MR-PRESSO and leave-one-out (LOO) analysis. In order to ensure the robustness of the causal association between exposure and outcome, the Bonferroni adjustment was employed. Additionally, we conducted analyses of the metabolic pathways of the identified metabolites using the Kyoto Encyclopedia of Genes and Genomes (KEGG) and the Small Molecule Pathway Database (SMPDB) database.

**Results:**

The MR analysis revealed a total of 27 metabolites (16 known and 11 unknown) causally linked to the development and progression of sepsis. After applying the Bonferroni correction, 3-carboxy-4-methyl-5-propyl-2-furanpropanoate (CMPF) remained significant in relation to 28-day all-cause mortality in sepsis. By pathway enrichment analysis, we identified four significant metabolic pathways. Notably, the Alpha Linolenic Acid and Linoleic Acid metabolism pathway emerged as a pivotal contributor to the occurrence and progression of sepsis.

**Conclusion:**

This study provides preliminary evidence of causal associations between human blood metabolites and sepsis, as ascertained by MR analysis. The findings offer valuable insights into the pathogenesis of sepsis and may provide insight into preventive and therapeutic approaches.

## Graphical abstract

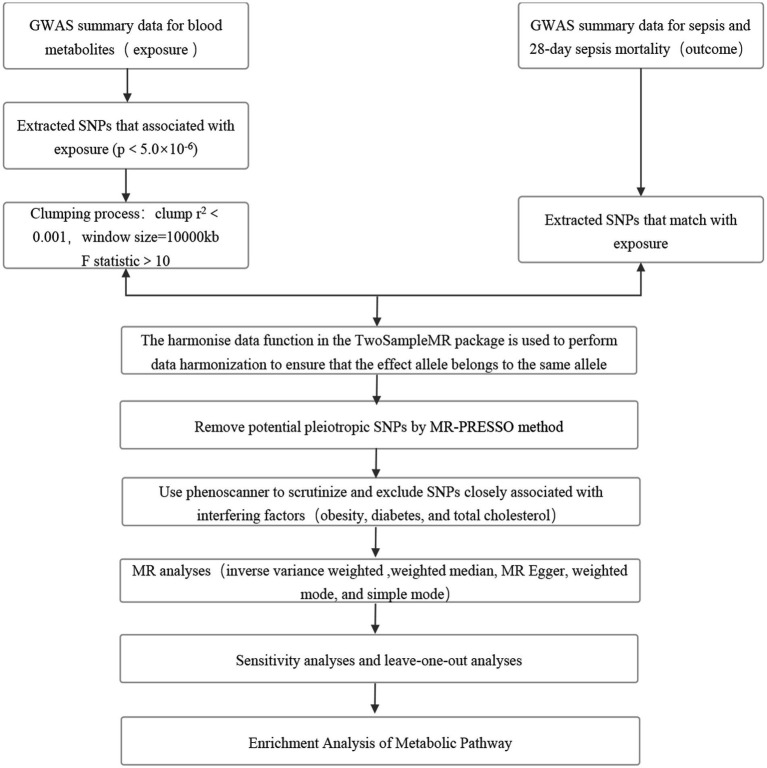

## 1 Introduction

As a result of a dysregulated host response to infection, sepsis can lead to life-threatening organ dysfunction with inflammation and immune dysfunction. This condition exhibits a significant mortality rate (1, 2). Globally, there were approximately 48.9 million cases of sepsis reported in 2017, resulting in an estimated 11 million cases of sepsis-related deaths, which accounts for 19.7% of global mortality (3). Clinicians often encounter challenges in identifying individuals at risk of sepsis due to possible infections. Identifying sepsis as a major concern for global health and patient safety, the World Health Organization (WHO) stresses the importance of recognizing the contributing factors that either elevate or lower the risk of sepsis (4).

In recent years, the discipline of metabolomics has surfaced as a methodical strategy to explore small molecule metabolites in living beings, providing fresh prospects to improve our comprehension of the fundamental processes implicated in the initiation and advancement of illnesses (5). Inflammation can manifest in aseptic forms, such as those resulting from surgical procedures or trauma, or infectious forms, such as sepsis (6). Metabolomics holds promise in offering valuable insights to support clinical decision-making. Importantly, the profiles of metabolites have shown the capacity to successfully differentiate sterile inflammation from sepsis in both human and animal studies (7, 8). Simultaneously, metabolite profiles hold the potential to provide reasonably accurate predictions regarding the occurrence and progression of sepsis. In neonates, metabolite profiles can differentiate between healthy individuals and those with sepsis, as well as reveal distinct patterns between early-onset and late-onset sepsis (9). In addition, following traumatic injury, in adult patients admitted to the intensive care unit (ICU), metabolite profiles can effectively distinguish between those who develop sepsis and those who do not (10). Metabolite profiles can also differentiate between the prognosis of septic patients, highlighting significant differences between survivors and non-survivors (11–13).

While several metabolites have been observed to be associated with sepsis in population-based cohorts (14), a systematic evaluation of the impact of blood metabolites on sepsis has not yet been conducted. Traditional studies face challenges in identifying and establishing potential causal relationships between blood metabolites and sepsis due to unavoidable confounding factors. MR analysis leverages genetic variants as instrumental variables (IVs) to mitigate confounding and assess the association between exposure and outcome, making it a widely used method for identifying reliable risk factors for various diseases (15). In contrast to observational studies, MR studies effectively minimize confounding variables and provide more robust causal evidence by utilizing natural random allocation (16, 17). As a result, the objective of this study is to utilize the strengths of the MR approach to investigate the correlation between blood metabolites and sepsis in a comprehensive way. The analysis will be based on extensive metabolomics data and clinical information. Statistical methods and genetic variant detection techniques will be employed to identify metabolites associated with sepsis and elucidate their potential contributions to the pathophysiological mechanisms underlying sepsis. Additional analyses will be performed to acquire more profound understanding of the function of these metabolites in sepsis.

## 2 Materials and methods

### 2.1 Data source

Blood metabolite data were obtained from the metabolomics Genome-wide association study (GWAS) server.^1^ Summary data from a previously published GWAS study on human blood metabolites were utilized (18), a total of 7,824 European participants participated in this study, including 1768 participants from the KORA F4 study conducted in Germany, and 6,056 participants from the UK Twin Study. This GWAS dataset represents the most comprehensive genetic loci information for 2.1 million Single Nucleotide Polymorphisms (SNPs) associated with 486 blood metabolites. Among these metabolites, 177 metabolites remain unidentified due to their unknown chemical identity, while 309 metabolites have been classified into eight broad metabolic groups: amino acids, carbohydrates, cofactors and vitamins, energy, lipids, nucleotides, peptides, and xenobiotics, relying on data from KEGG (19).

Sepsis data were obtained from the IEU OpenGWAS project,^2^ including sepsis and sepsis-related 28-day all-cause mortality. These data derive from a European population consisting of 486,484 participants drawn from the UK Biobank (20). Among them, 11,643 individuals had sepsis, and a total of 12,243,539 SNPs were considered. Within this group, 1,896 individuals succumbed to all causes within 28 days, while survivors were used as controls, involving a total of 12,243,487 SNPs.

### 2.2 Study design

**Figure 1:**
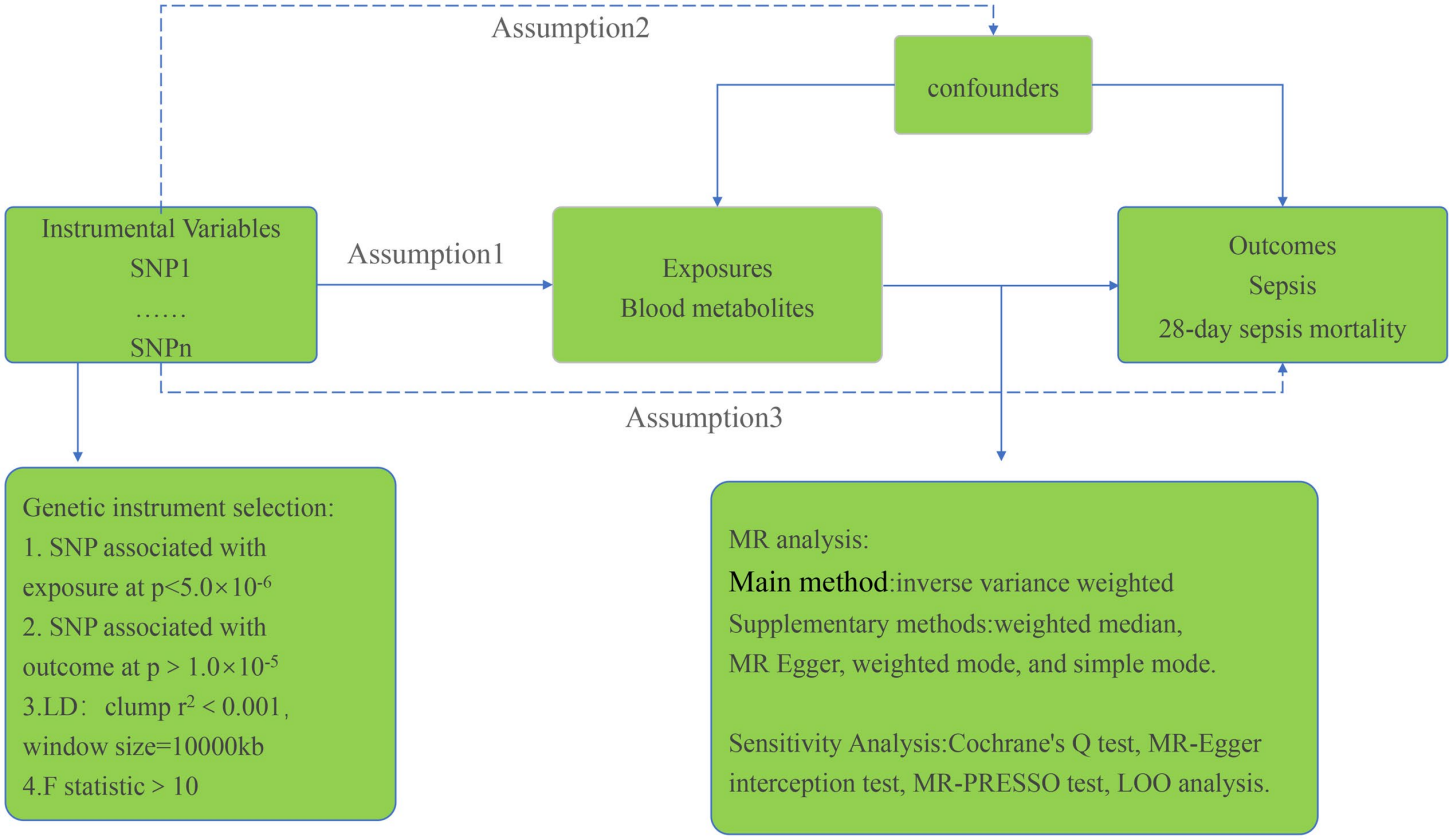
Study design overview and assumptions of the MR design. Notes: SNP: single nucleotide polymorphism, LD: linkage disequilibrium, IVW: inverse-variance weighted, MR-PRESSO: Mendelian randomization pleiotropy residual sum and outlier.

MR was used to investigate the relationship between blood metabolites and sepsis, as well as sepsis-related mortality. The MR analysis conducted in this study adhered to three key assumptions (Figure 1): (1) Strong association between exposure factors and IVs; (2) Absence of confounding factors associated with the IVs; (3) IVs chosen did not have a direct impact on the outcome but influenced it solely through exposure factors (21).

### 2.3 Genetic instrument selection

The selection of IVs for analysis required a robust association with the exposure factor. To ensure precision and efficacy in establishing causal connections between blood metabolites and sepsis risk, SNPs with *p*-values below 5 × 10^−6^, representing locus-wide significance, were chosen. Furthermore, selected instrumental variables needed to pass independence tests successfully, setting the linkage disequilibrium parameter (*R*^2^) of SNPs to 0.001 and the genetic distance to 10,000 kb. Additionally, IVs with *F* values <10 were excluded to ensure the strength of the association between IVs and exposure factors (22). IVs having *p*-values below 1.0 × 10^−5^ concerning the outcome were also disregarded. The Phenoscanner software package was employed to identify covariates associated with potential confounding factors like obesity, diabetes, and total cholesterol to prevent these factors from confounding the impact of exposure on the outcome. Significant associations (*p* < 1.0 × 10^−5^) between SNPs and potential confounding factors led to the exclusion of those SNPs from the analysis. Subsequent MR analysis was conducted to validate the results’ strength. MR analysis was specifically carried out on metabolites with at least 3 SNPs (23).

### 2.4 Statistical analysis

The inverse variance weighted (IVW) method was employed as the primary analysis method in this study to assess the significant causal relationship between metabolites and sepsis, as well as 28-day all-cause mortality in sepsis (*p* < 0.05). Furthermore, several other MR analysis methods, including weighted median, MR Egger, weighted mode, and simple mode, were used as complementary approaches. Analysis was conducted only on metabolites exhibiting consistent associations across all five methods. To assess heterogeneity and pleiotropy in the IVW method, both Cochran’s Q test and MR-Egger intercept analysis were performed. Metabolites exhibiting pleiotropy in the IVW analysis (*p* < 0.05) were removed, and IVs were subjected to MR-PRESSO (version 1.0) to identify and eliminate outlier SNPs, addressing horizontal pleiotropy. LOO analysis was conducted to confirm that MR findings were not influenced by individual SNPs. The study focused on sepsis and 28-day mortality as outcomes, identifying core metabolites associated with the incidence and progression of sepsis. Given the numerous MR analyses during the metabolite screening process, Bonferroni adjustment was applied to rigorously evaluate the causal relationships of identified metabolites (*p* < 0.05/309 = 0.000167). Using odds ratios and 95% confidence intervals, we estimated causal effects concerning the association between blood metabolites and sepsis risk and mortality. The statistical analyses were carried out using R (version 4.3.0).

### 2.5 Enrichment analysis of metabolic pathway

To gain further insights into metabolic pathways associated with sepsis occurrence and progression, MetaboAnalyst 5.0^3^ was utilized for metabolic pathway analysis. First, we identified the corresponding ID of these metabolites in the MetaboAnalyst 5.0. Then, we used Pathway Analysis modules in the Annotated Features mode to perform the pathway analysis, relying on the KEGG and SMPDB databases. This analysis was performed following all the specified conditions for MR analysis.

## 3 Results

A thorough IV screening was performed for each of the 486 metabolites. Following the IV selection criteria, we included a comprehensive set of 5,538 SNPs related to sepsis in the analysis, ensuring that each metabolite was associated with a minimum of 3 SNPs. After scrutinizing Phenoscanner and excluding SNPs strongly correlated with confounding factors, a total of 17 SNPs were excluded from the analysis. Detailed information about the selected IVs in Tables S1, S2 in Supplementary material. Scatter plots and funnel plots illustrating the MR analysis results are presented in Figures S3–S6 in Supplementary material.

### 3.1 Two samples MR analysis

**Figure 2:**
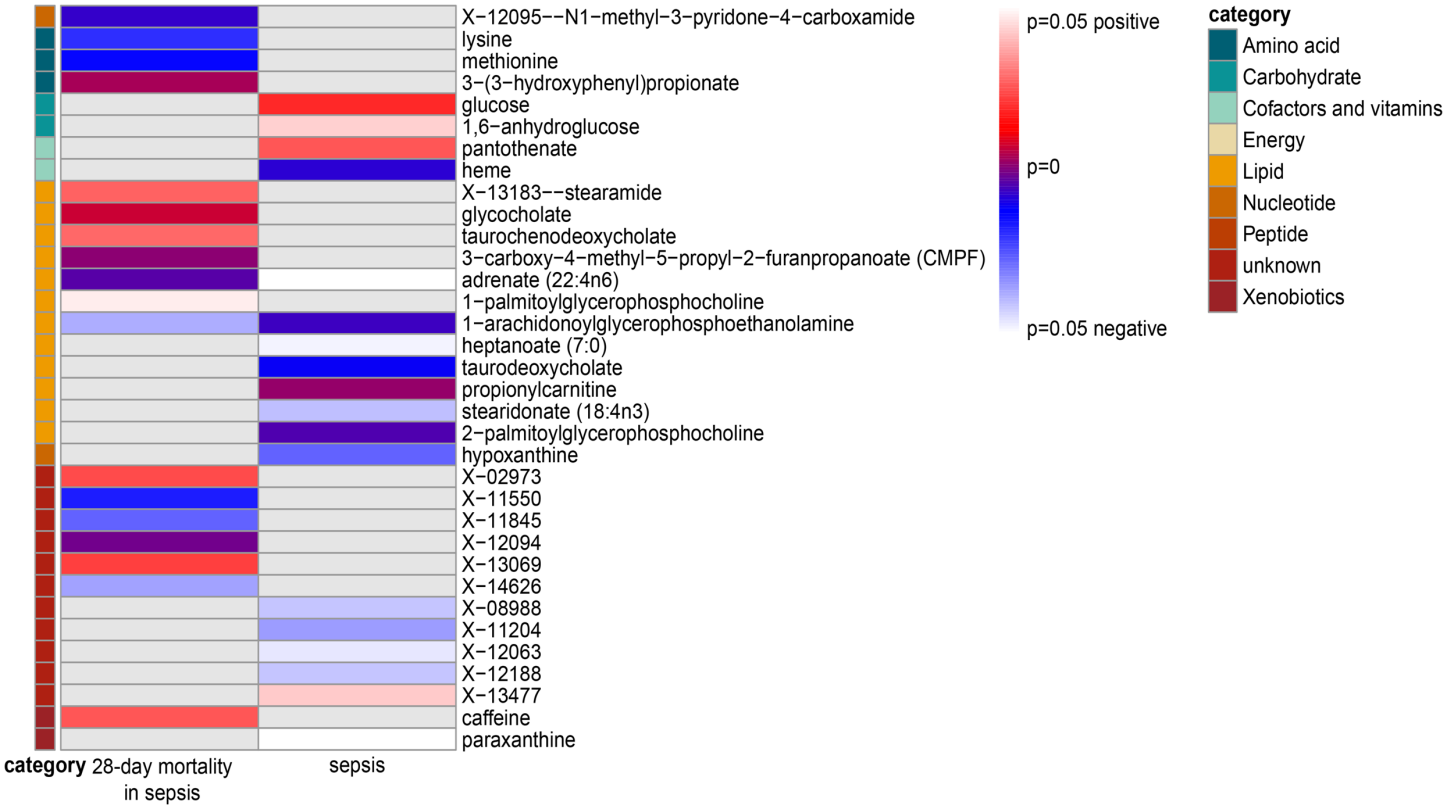
Heatmap of the IVW analysis results for sepsis and 28-day mortality in sepsis. Note: Heatmaps showing the *p*-values of the IVW analysis and the direction of the result.

The IVW analysis was employed to screen metabolites, focusing on their associations with sepsis and 28-day all-cause mortality. Out of the initial pool, 34 blood metabolites were selected based on a significance threshold of *p* < 0.05. A visualization of these metabolites was displayed in a heatmap (Figure 2). Subsequently, these 34 metabolites underwent four other types of MR analysis. We selected metabolites that consistently showed significant associations across all five methods. Ultimately, 27 blood metabolites were identified that exhibited a causal relationship with the risk of sepsis and 28-day all-cause mortality in sepsis.

#### 3.1.1 Sepsis

**Figure 3:**
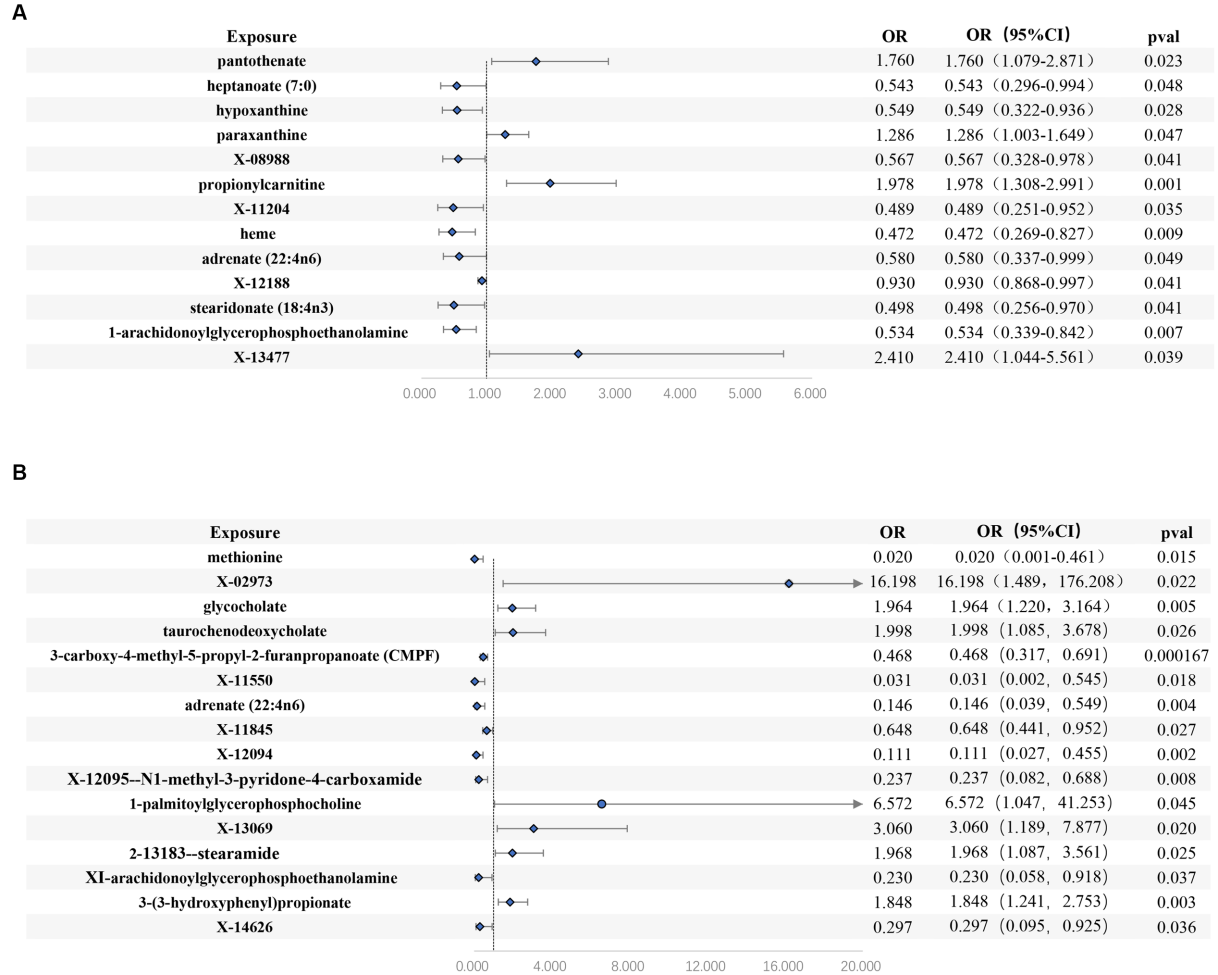
Forest plots of estimates identified with inverse variance weighted. **(A)** sepsis, **(B)** 28-day death in sepsis.

We found 13 causal relationships between blood metabolites and sepsis risk in this study (Figure 3 and Table 1). Among these, pantothenate, paraxanthine, propionylcarnitine, X-13477 were associated with a higher risk, whereas heptanoate, hypoxanthine, X-08988, X-11204, heme, Adrenate (22:4n6), X-12188, stearidonate, and 1-arachidonoylglycerophosphoethanolamine were protective factors. The Cochrane’s Q test did not indicate any statistically significant heterogeneity (*p* > 0.05). Furthermore, both the MR-Egger interception test and MR-PRESSO test did not reveal any signs of pleiotropy (p > 0.05). Lastly, the LOO analysis (Figure S1 in Supplementary material) demonstrated that after systematically excluding each SNP, pantothenate, propionylcarnitine, and 1-arachidonoylglycerophosphoethanolamine consistently yielded stable results.

**Table 1:** MR results and sensitivity analysis of blood metabolites on sepsis.

Level	Exposure	Outcome	Method	Nsnps	Se	P	OR (95% CI)	Cochran’s Q *p*	MR-Egger intercept *p*	MR-PRESSO global test *p*
Cofactors and vitamins	Pantothenate	Sepsis	Inverse variance weighted	15	0.250	0.023	1.760 (1.079 ~ 2.871)	0.983	0.299	0.985
			MR Egger	15	0.470	0.778	1.145 (0.456 ~ 2.875)			
			Simple mode	15	0.556	0.364	1.685 (0.567 ~ 5.010)			
			Weighted median	15	0.351	0.098	1.787 (0.899 ~ 3.555)			
			Weighted mode	15	0.497	0.260	1.794 (0.677 ~ 4.755)			
Lipid	Heptanoate (7:0)	Sepsis	Inverse variance weighted	20	0.309	0.048	0.543 (0.296 ~ 0.994)	0.698	0.951	0.718
			MR Egger	20	0.919	0.552	0.573 (0.094 ~ 3.472)			
			Simple mode	20	0.708	0.488	0.606 (0.151 ~ 2.428)			
			Weighted median	20	0.444	0.183	0.554 (0.232 ~ 1.322)			
			Weighted mode	20	0.596	0.345	0.561 (0.174 ~ 1.805)			
Nucleotide	Hypoxanthine	Sepsis	Inverse variance weighted	13	0.272	0.028	0.549 (0.322 ~ 0.936)	0.412	0.752	0.460
			MR Egger	13	0.780	0.650	0.695 (0.151 ~ 3.209)			
			Simple mode	13	0.608	0.211	0.448 (0.136 ~ 1.474)			
			Weighted median	13	0.380	0.068	0.500 (0.238 ~ 1.053)			
			Weighted mode	13	0.506	0.152	0.461 (0.171 ~ 1.242)			
Xenobiotics	Paraxanthine	Sepsis	Inverse variance weighted	7	0.127	0.047	1.286 (1.003 ~ 1.649)	0.603	0.973	0.567
			MR Egger	7	0.427	0.560	1.305 (0.565 ~ 3.011)			
			Simple mode	7	0.296	0.680	1.137 (0.636 ~ 2.033)			
			Weighted median	7	0.181	0.289	1.212 (0.850 ~ 1.728)			
			Weighted mode	7	0.206	0.875	1.035 (0.690 ~ 1.550)			
unknown	X-08988	Sepsis	Inverse variance weighted	21	0.279	0.041	0.567 (0.328 ~ 0.978)	0.309	0.517	0.367
			MR Egger	21	0.715	0.177	0.367 (0.090 ~ 1.491)			
			Simple mode	21	0.697	0.699	0.760 (0.194 ~ 2.982)			
			Weighted median	21	0.421	0.203	0.585 (0.257 ~ 1.334)			
			Weighted mode	21	0.491	0.386	0.647 (0.247 ~ 1.694)			
Lipid	Propionylcarnitine	Sepsis	Inverse variance weighted	24	0.211	0.001	1.978 (1.308 ~ 2.991)	0.679	0.770	0.699
			MR Egger	24	0.462	0.096	2.233 (0.904 ~ 5.521)			
			Simple mode	24	0.479	0.134	2.102 (0.823 ~ 5.372)			
			Weighted median	24	0.293	0.006	2.236 (1.258 ~ 3.972)			
			Weighted mode	24	0.374	0.055	2.134 (1.024 ~ 4.445)			
unknown	X-11204	Sepsis	Inverse variance weighted	24	0.340	0.035	0.489 (0.251 ~ 0.952)	0.556	0.952	0.589
			MR Egger	24	1.275	0.620	0.527 (0.043 ~ 6.415)			
			Simple mode	24	0.948	0.252	0.328 (0.051 ~ 2.105)			
			Weighted median	24	0.485	0.030	0.349 (0.135 ~ 0.903)			
			Weighted mode	24	0.898	0.219	0.321 (0.055 ~ 1.868)			
Cofactors and vitamins	heme*	Sepsis	Inverse variance weighted	5	0.286	0.009	0.472 (0.269 ~ 0.827)	0.344	0.254	0.414
			MR Egger	5	0.983	0.124	0.125 (0.018 ~ 0.857)			
			Simple mode	5	0.550	0.163	0.391 (0.133 ~ 1.149)			
			Weighted median	5	0.381	0.035	0.447 (0.212 ~ 0.943)			
			Weighted mode	5	0.497	0.149	0.412 (0.156 ~ 1.092)			
Lipid	adrenate (22:4n6)	Sepsis	Inverse variance weighted	5	0.277	0.049	0.580 (0.337 ~ 0.999)	0.979	0.900	0.987
			MR Egger	5	1.018	0.553	0.508 (0.069 ~ 3.734)			
			Simple mode	5	0.415	0.220	0.548 (0.243 ~ 1.234)			
			Weighted median	5	0.338	0.077	0.55 (0.284 ~ 1.067)			
			Weighted mode	5	0.364	0.170	0.544 (0.266 ~ 1.111)			
unknown	X-12188	Sepsis	Inverse variance weighted	11	0.035	0.041	0.930 (0.868 ~ 0.997)	0.496	0.821	0.536
			MR Egger	11	0.075	0.271	0.916 (0.791 ~ 1.060)			
			Simple mode	11	0.074	0.827	0.984 (0.851 ~ 1.137)			
			Weighted median	11	0.048	0.448	0.964 (0.877 ~ 1.060)			
			Weighted mode	11	0.045	0.376	0.959 (0.878 ~ 1.048)			
Lipid	stearidonate (18:4n3)	Sepsis	Inverse variance weighted	5	0.340	0.041	0.498 (0.256 ~ 0.970)	0.214	0.830	0.333
			MR Egger	5	1.361	0.793	0.676 (0.047 ~ 9.737)			
			Simple mode	5	0.493	0.135	0.398 (0.151 ~ 1.045)			
			Weighted median	5	0.352	0.013	0.417 (0.209 ~ 0.832)			
			Weighted mode	5	0.389	0.081	0.406 (0.189 ~ 0.869)			
Lipid	1-arachidonoylglycerophosphoethanolamine*	Sepsis	Inverse variance weighted	14	0.232	0.007	0.534 (0.339 ~ 0.842)	0.996	0.829	1.000
			MR Egger	14	0.602	0.237	0.473 (0.145 ~ 1.538)			
			Simple mode	14	0.467	0.152	0.491 (0.196 ~ 1.228)			
			Weighted median	14	0.318	0.027	0.496 (0.266 ~ 0.925)			
			Weighted mode	14	0.366	0.074	0.491 (0.240 ~ 1.007)			
unknown	X-13477	Sepsis	Inverse variance weighted	4	0.427	0.039	2.410 (1.044 ~ 5.561)	0.980	0.827	0.972
			MR Egger	4	0.993	0.382	3.013 (0.43 ~ 21.088)			
			Simple mode	4	0.623	0.314	2.120 (0.625 ~ 7.189)			
			Weighted median	4	0.494	0.083	2.356 (0.895 ~ 6.202)			
			Weighted mode	4	0.554	0.269	2.114 (0.714 ~ 6.260)			

CI, confidence interval; NSNPs, number of single nucleotide polymorphisms.

#### 3.1.2 28-Day mortality in sepsis

We identified 16 causal relationships between blood metabolites and 28-day all-cause mortality in patients with sepsis identified (Figure 3 and Table 2). Among these, X-02973, glycocholate, taurochenodeoxycholate, 1-palmitoylglycerophosphocholine, X-13069, stearamide, 3-(3-hydroxyphenyl) propionate were risk factors, while methionine, 3-carboxy-4-methyl-5-propyl-2-furanpropanoate (CMPF), X-11550, Adrenate (22:4n6), X-11845, X-12094, N1-methyl-3-pyridone-4-carboxamide, 1-arachidonoylglycerophosphoethanolamine, X-14626 were protective factors. Similar to the sepsis analysis, the Cochrane’s Q test did not indicate any statistically significant heterogeneity (*p* > 0.05). Moreover, both the MR-Egger interception test and MR-PRESSO test did not detect any signs of pleiotropy (p > 0.05). The LOO analysis (Figure S2 in Supplementary material) demonstrated that after systematically excluding each SNP, methionine, glycocholate, and CMPF consistently yielded stable results.

**Table 2:** MR results and sensitivity analysis of blood metabolites on 28-day death in sepsis.

Level	Exposure	Outcome	Method	Nsnps	Se	*p*	OR (95% CI)	Cochran’s Q *p*	MR-Egger intercept *p*	MR-PRESSO global test *p*
Amino acid	methionine	Sepsis (28 day death)	Inverse variance weighted	12	1.605	0.015	0.020 (0.001 ~ 0.461)	0.921	0.517	0.919
			MR Egger	12	4.945	0.878	0.459 (0.001 ~ 7434.500)			
			Simple mode	12	3.251	0.562	0.143 (0.001 ~ 83.902)			
			Weighted median	12	2.244	0.317	0.106 (0.001 ~ 8.624)			
			Weighted mode	12	2.886	0.613	0.223 (0.001 ~ 63.648)			
unknown	X-02973	Sepsis (28 day death)	Inverse variance weighted	16	1.218	0.022	16.198 (1.489 ~ 176.208)	0.792	0.719	0.797
			MR Egger	16	5.641	0.408	122.456 (0.002 ~ 7760268.183)			
			Simple mode	16	2.726	0.194	40.819 (0.195 ~ 8538.371)			
			Weighted median	16	1.588	0.029	31.860 (1.417 ~ 716.415)			
			Weighted mode	16	2.674	0.212	32.611 (0.173 ~ 6155.931)			
Lipid	glycocholate	Sepsis (28 day death)	Inverse variance weighted	6	0.243	0.005	1.964 (1.220 ~ 3.164)	0.885	0.961	0.879
			MR Egger	6	0.333	0.108	1.988 (1.035 ~ 3.820)			
			Simple mode	6	0.506	0.154	2.341 (0.868 ~ 6.315)			
			Weighted median	6	0.336	0.048	1.942 (1.005 ~ 3.752)			
			Weighted mode	6	0.325	0.115	1.857 (0.981 ~ 3.515)			
Lipid	taurochenodeoxycholate	Sepsis (28 day death)	Inverse variance weighted	6	0.311	0.026	1.998 (1.085 ~ 3.678)	0.489	0.863	0.662
			MR Egger	6	0.563	0.240	2.174 (0.722 ~ 6.549)			
			Simple mode	6	0.598	0.413	1.706 (0.528 ~ 5.504)			
			Weighted median	6	0.414	0.138	1.847 (0.821 ~ 4.154)			
			Weighted mode	6	0.393	0.162	1.906 (0.883 ~ 4.116)			
Lipid	3-carboxy-4-methyl-5-propyl-2-furanpropanoate (CMPF)	Sepsis (28 day death)	Inverse variance weighted	10	0.199	0.000	0.468 (0.317 ~ 0.691)	0.471	0.780	0.602
			MR Egger	10	0.505	0.115	0.410 (0.152 ~ 1.102)			
			Simple mode	10	0.388	0.042	0.400 (0.187 ~ 0.855)			
			Weighted median	10	0.265	0.000	0.397 (0.236 ~ 0.667)			
			Weighted mode	10	0.345	0.027	0.403 (0.205 ~ 0.793)			
unknown	X-11550	Sepsis (28 day death)	Inverse variance weighted	15	1.465	0.018	0.031 (0.002 ~ 0.545)	0.262	0.770	0.304
			MR Egger	15	5.560	0.378	0.006 (0.001 ~ 337.742)			
			Simple mode	15	3.457	0.156	0.006 (0.001 ~ 4.943)			
			Weighted median	15	1.900	0.022	0.013 (0.001 ~ 0.540)			
			Weighted mode	15	3.208	0.114	0.004 (0.001 ~ 2.393)			
Lipid	adrenate (22:4n6)	Sepsis (28 day death)	Inverse variance weighted	5	0.676	0.004	0.146 (0.039 ~ 0.549)	0.633	0.306	0.729
			MR Egger	5	2.482	0.145	0.008 (0.001 ~ 1.001)			
			Simple mode	5	1.160	0.174	0.148 (0.015 ~ 1.433)			
			Weighted median	5	0.831	0.012	0.123 (0.024 ~ 0.629)			
			Weighted mode	5	0.949	0.087	0.118 (0.018 ~ 0.755)			
unknown	X-11845	Sepsis (28 day death)	Inverse variance weighted	11	0.196	0.027	0.648 (0.441 ~ 0.952)	0.374	0.389	0.421
			MR Egger	11	0.471	0.922	0.954 (0.379 ~ 2.402)			
			Simple mode	11	0.454	0.111	0.452 (0.185 ~ 1.101)			
			Weighted median	11	0.274	0.148	0.672 (0.393 ~ 1.151)			
			Weighted mode	11	0.387	0.266	0.633 (0.297 ~ 1.353)			
unknown	X-12094	Sepsis (28 day death)	Inverse variance weighted	5	0.721	0.002	0.111 (0.027 ~ 0.455)	0.554	0.211	0.569
			MR Egger	5	2.361	0.092	0.003 (0.001 ~ 0.320)			
			Simple mode	5	1.359	0.146	0.087 (0.006 ~ 1.244)			
			Weighted median	5	0.938	0.008	0.084 (0.013 ~ 0.530)			
			Weighted mode	5	1.169	0.076	0.062 (0.006 ~ 0.616)			
Nucleotide	X-12095--N1-methyl-3-pyridone-4-carboxamide	Sepsis (28 day death)	Inverse variance weighted	15	0.543	0.008	0.237 (0.082 ~ 0.688)	0.620	0.671	0.604
			MR Egger	15	1.158	0.406	0.370 (0.038 ~ 3.582)			
			Simple mode	15	1.499	0.263	0.174 (0.009 ~ 3.283)			
			Weighted median	15	0.803	0.100	0.267 (0.055 ~ 1.290)			
			Weighted mode	15	1.206	0.541	0.469 (0.044 ~ 4.991)			
Lipid	1-palmitoylglycerophosphocholine	Sepsis (28 day death)	Inverse variance weighted	20	0.937	0.045	6.572 (1.047 ~ 41.253)	0.764	0.617	0.780
			MR Egger	20	4.305	0.363	55.687 (0.012 ~ 257066.749)			
			Simple mode	20	2.523	0.133	52.689 (0.375 ~ 7401.713)			
			Weighted median	20	1.322	0.013	26.630 (1.994 ~ 355.648)			
			Weighted mode	20	2.473	0.133	48.505 (0.381 ~ 6172.563)			
unknown	X-13069	Sepsis (28 day death)	Inverse variance weighted	11	0.482	0.020	3.060 (1.189 ~ 7.877)	0.667	0.349	0.668
			MR Egger	11	1.497	0.127	12.416 (0.660 ~ 233.493)			
			Simple mode	11	1.095	0.584	1.857 (0.217 ~ 15.872)			
			Weighted median	11	0.645	0.222	2.200 (0.621 ~ 7.785)			
			Weighted mode	11	1.116	0.550	1.994 (0.224 ~ 17.772)			
Lipid	X-13183--stearamide	Sepsis (28 day death)	Inverse variance weighted	9	0.303	0.025	1.968 (1.087 ~ 3.561)	0.602	0.520	0.628
			MR Egger	9	0.639	0.142	2.880 (0.823 ~ 10.078)			
			Simple mode	9	0.688	0.409	1.820 (0.473 ~ 7.007)			
			Weighted median	9	0.434	0.230	1.684 (0.719 ~ 3.944)			
			Weighted mode	9	0.578	0.535	1.454 (0.469 ~ 4.513)			
Lipid	1-arachidonoylglycerophosphoethanolamine*	Sepsis (28 day death)	Inverse variance weighted	14	0.706	0.037	0.230 (0.058 ~ 0.918)	0.091	0.418	0.127
			MR Egger	14	1.852	0.143	0.055 (0.001 ~ 2.070)			
			Simple mode	14	1.707	0.093	0.045 (0.002 ~ 1.285)			
			Weighted median	14	0.782	0.002	0.093 (0.020 ~ 0.430)			
			Weighted mode	14	0.902	0.019	0.090 (0.015 ~ 0.528)			
Amino acid	3-(3-hydroxyphenyl)propionate	Sepsis (28 day death)	Inverse variance weighted	7	0.203	0.003	1.848 (1.241 ~ 2.753)	0.411	0.871	0.424
			MR Egger	7	0.762	0.549	1.632 (0.366 ~ 7.268)			
			Simple mode	7	0.495	0.114	2.499 (0.947 ~ 6.595)			
			Weighted median	7	0.289	0.004	2.317 (1.316 ~ 4.080)			
			Weighted mode	7	0.443	0.074	2.609 (1.095 ~ 6.217)			
unknown	X-14626	Sepsis (28 day death)	Inverse variance weighted	10	0.580	0.036	0.297 (0.095 ~ 0.925)	0.749	0.948	0.799
			MR Egger	10	1.150	0.298	0.278 (0.029 ~ 2.646)			
			Simple mode	10	1.163	0.320	0.294 (0.003 ~ 2.876)			
			Weighted median	10	0.773	0.091	0.271 (0.060 ~ 1.232)			
			Weighted mode	10	0.802	0.143	0.276 (0.057 ~ 1.330)			

CI, confidence interval; NSNPs, number of single nucleotide polymorphisms.

### 3.2 Bonferroni-corrected test and analysis of metabolic pathway enrichment

After applying the Bonferroni correction, we observed that CMPF maintained its significance in relation to 28-day all-cause mortality in sepsis (*p* < 0.000167). By incorporating data from the KEGG and SMPDB databases, the MR analysis identified four significant pathways (Table 3). Notably, the Alpha Linolenic Acid and Linoleic Acid Metabolism pathway played a crucial role in the occurrence and progression of sepsis.

**Table 3:** Results of pathway enrichment analysis.

Metabolic pathway	Trait	Database	*p*
Caffeine metabolism	sepsis	KEGG	0.031885
alpha-Linolenic acid metabolism	sepsis	KEGG	0.04129
Alpha linolenic acid and linoleic acid metabolism	sepsis	SMPBD	0.0047302
	sepsis 28	SMPBD	0.046867
Primary bile acid biosynthesis	sepsis 28	KEGG	0.0025375

KEGG, Kyoto Encyclopedia of Genes and Genomes; SMPDB, Small Molecule Pathway Database.

## 4 Discussion

This study integrated two large GWAS datasets and used a rigorous MR design to investigate the causal associations between 486 blood metabolites and sepsis. Our analysis revealed that 13 blood metabolites exhibited a causal relationship with sepsis, while 16 blood metabolites demonstrated a causal relationship with 28-day mortality in sepsis. Specifically, we observed that 1-arachidonoylglycerophosphoethanolamine and Adrenate (22:4n6) were both significantly associated with the occurrence and progression of sepsis. After Bonferroni correction testing, CMPF showed a strong causal relationship with lower 28-day mortality in sepsis. These results have the potential to furnish valuable indications for identifying early diagnostic biomarkers and potential therapeutic targets for sepsis.

1-Arachidonoylglycerophosphoethanolamine is a novel metabolite related to the endogenous cannabinoid system, producing arachidonoylethanolamine (anandamide, AEA) through the cleavage of glycerophosphoethanolamine by phosphodiesterase. AEA, an endogenous cannabinoid, is synthesized by macrophages in response to pathological conditions like shock and is considered a pathogenic mediator in septic shock development (24). Studies on animal models have confirmed AEA’s role in regulating the immune system, exhibiting various effects in sepsis, including anti-inflammatory, antioxidant, pro-apoptotic, and immunomodulatory effects (25–27). Activation of AEA receptors on immune cells reduces pro-inflammatory cytokine secretion and the recruitment of neutrophils and macrophages (28, 29). Recent research findings suggest that low baseline plasma AEA levels may serve as prognostic indicators for septic patients requiring prolonged mechanical ventilation. Furthermore, a lower concentration of AEA has been identified as a prognostic factor for hospital stays exceeding 10 days (30). In another study, AEA was found to attenuate acute respiratory distress syndrome induced by Staphylococcus Enterotoxin B by suppressing inflammation through the down-regulation of key miRNA that regulates immunosuppressive pathways (31). These findings imply that the endocannabinoid AEA may possess a protective effect against severe inflammation and could potentially be utilized in the management of sepsis cases with multiple complications. This study highlights that 1-arachidonoylglycerophosphoethanolamine exhibits a protective effect against sepsis, indicating its potential as a novel and promising therapeutic target for sepsis treatment.

Adrenate (22:4n6) has been identified as a protective factor against lacunar stroke in a previous MR study (32). Dihomo-isofurans (dihomo-IsoPs), which are peroxidation products derived from Adrenate (22:4n6), play a crucial role in the composition of white matter. These compounds hold potential as selective biomarkers for quantifying *in vivo* free radical damage to neuronal membranes Moreover, plasma biomarkers associated with Adrenate (22:4n6) and its derivatives hold promise for early and differential diagnosis of Alzheimer’s disease (33). While current research on Adrenate (22:4n6) and its derivatives primarily focuses on neurodegenerative diseases (34), our study has revealed its potential significance in the development and progression of sepsis. However, the underlying mechanisms of this phenomenon remain unknown as our investigation primarily focused on correlation analysis. Therefore, further research is essential to explore and elucidate these mechanistic explanations.

The present study conducted a comprehensive MR analysis, revealing a robust causal association between CMPF and reduced mortality from sepsis within a 28-day period. In individuals who consume fish and fish oil as well as polyunsaturated fatty acids through high-temperature cooking, CMPF, a metabolite produced from furan fatty acids, is found in higher concentrations (35). Numerous investigations have established a correlation between CMPF and the development of type 2 diabetes, while recent research has indicated that serum CMPF levels are inversely associated with the risk of type 2 diabetes (36–38). Given that diabetes mellitus is a recognized risk factor for the onset of sepsis, the inverse relationship between CMPF and diabetes mellitus could potentially serve as a significant protective factor. Additionally, a study on periodontitis has shown that elevated CMPF levels are linked to a reduced occurrence of gingival inflammation and a less severe form of periodontitis (39). Moreover, CMPF has exhibited potential anti-inflammatory properties. For instance, an extract derived from green-lipped mussels, known for their furan fatty acid content, has demonstrated promising outcomes in alleviating symptoms associated with rheumatoid arthritis in patients. This therapeutic effect is achieved through the reduction of interleukin (IL) 1β, prostaglandin (PGE2), and tumor necrosis factor α (TNF-α) levels (40). Furthermore, in experiments with mice, CMPF treatment resulted in improved fat removal from the liver and reduced fat storage, effectively preventing lipid buildup in the liver and the onset of hepatic insulin resistance induced by a high-fat diet (41). Sepsis patients, particularly those with obesity, often exhibit insulin resistance (42). Empirical research has confirmed that hyperglycemia in sepsis patients is associated with an unfavorable prognosis. Therefore, it is imperative to maintain sepsis patients’ blood glucose levels within a reasonable range. Elevated levels of CMPF have the potential to enhance insulin sensitivity, mitigate lipid accumulation, and counteract insulin resistance induced by a high-fat diet. This, in turn, can effectively regulate blood glucose levels in sepsis patients and improve their prognosis (43).

The analysis of metabolic pathways in sepsis and the 28-day all-cause mortality has revealed a significant elevation in the value of Alpha Linolenic Acid (ALA) and Linoleic Acid (LA) metabolism. ALA and LA are crucial constituents of Omega-3 and Omega-6 polyunsaturated fatty acids. Additionally, they can undergo conversion in the body to form longer-chain Omega-3 and Omega-6 polyunsaturated fatty acids (44). The involvement of these fatty acid-derived metabolites holds significant importance in the development of sepsis. ALA, classified as an Omega-3 polyunsaturated fatty acid, has recently been identified as having the potential to mitigate sepsis-induced intestinal damage through several mechanisms, including the downregulation of miR-1-3p, increased expression of Notch3, and inhibition of the Smad pathway activation (45). Additionally, ALA possesses anti-inflammatory properties and can impede platelet aggregation and thrombus formation (46). LA, on the other hand, is an Omega-6 polyunsaturated fatty acid, and one of its significant metabolic byproducts is arachidonic acid (ARA) (47). ARA can undergo metabolism via the cyclooxygenase (COX) pathway, resulting in the production of prostaglandins (PGs) and thromboxanes (TXs). Additionally, ARA can be converted into leukotrienes (LTs) and lipoxins (LX) through the lipoxygenase (LOX) pathway. Consequently, ARA assumes a pro-inflammatory function within the inflammatory process, thereby facilitating the sequential progression of inflammation (47–49). In patients with sepsis, ARA levels are typically elevated, and this elevation is associated with increased inflammation (50). A recent MR study provided evidence supporting our hypothesis that omega-3 intake is associated with a lower risk of sepsis, while an elevation in the omega-6/omega-3 ratio is associated with a higher risk of sepsis-related mortality (51). Therefore, considering dietary supplementation or adjustments in ALA and LA intake is crucial in mitigating the risk of sepsis and its associated mortality. Further investigation into the intricate mechanisms through which these fatty acids and their metabolites operate in sepsis is warranted to unveil innovative clinical treatment and prevention strategies.

Our study offers several advantages. Firstly, it is the first investigation to examine the causal association between blood metabolites and the occurrence and progression of sepsis using MR analysis. MR is a statistical technique grounded in whole-genome sequencing data, employed to reveal causal relationships. It effectively minimizes bias and yields more reliable outcomes compared to conventional observational studies, such as randomized controlled trials. The identified causal associations may provide potential blood metabolites for subsequent mechanistic investigations. Secondly, the SNPs linked to blood metabolites were obtained from the most extensive and comprehensive GWAS meta-analysis conducted to date. Thirdly, our selection criteria for IVs were more rigorous compared to other studies, ensuring the reliability of our research. Moreover, the large sample size enhances statistical power, and rigorous sensitivity analysis ensures the robustness of our findings. In spite of this, our study also has limitations. Firstly, all study participants are of European ancestry, so caution is necessary when extrapolating the results to other populations. Furthermore, there were 11 unknown blood metabolites in the preliminary analysis, necessitating further research to explore their specific associations with sepsis. Lastly, while MR analysis is effective in etiological research, the metabolites causally associated with sepsis identified in this study require further experimental validation and exploration of their specific mechanisms. Therefore, further refinement of our study in this area is needed.

## 5 Conclusion

In this MR study, we successfully identified 13 blood metabolites that exhibit a causal relationship with sepsis and 16 blood metabolites associated with a causal relationship to 28-day all-cause mortality in sepsis. The identification of these blood metabolites, whether beneficial or detrimental, holds significant promise for enhancing our understanding of sepsis etiology and informing the development of preventive and therapeutic approaches for managing sepsis.

## Data availability statement

The original contributions presented in the study are included in the article/Supplementary material, further inquiries can be directed to the corresponding author.

## Author contributions

ZZ: Writing – original draft, Writing – review & editing, Conceptualization, Data curation. YY: Data curation, Writing – original draft. TC: Data curation, Writing – original draft. JY: Writing – original draft. WZ: Writing – original draft. YZ: Writing – original draft. YR: Writing – original draft. HW: Writing – original draft. XC: Writing – original draft. XZ: Conceptualization, Resources, Supervision, Writing – original draft, Writing – review & editing.

## Footnotes

^1^ https://metabolomics.helmholtz-muenchen.de/gwas/

^2^ https://gwas.mrcieu.ac.uk/

^3^ https://www.metaboanalyst.ca/
