# Supplementary material for: Investigating the impact of human blood metabolites on the Sepsis development and progression: a study utilizing two-sample Mendelian randomization
Source: Front Med (Lausanne). 2023 Dec 8;10:1310391. doi: 10.3389/fmed.2023.1310391 (PMC10748392; doi:10.3389/fmed.2023.1310391)
Supplement: Supplementary file 1 [file Data_Sheet_1.zip › Supplementary materials.docx]

**Supplementary Table S1:** **Selected instrumental variables and harmonization data of** **blood metabolites on sepsis.**

Notes: IVs: Instrumental Variables, MR Analysis: Mendelian Randomization Analysis.

**Supplementary Table S2: Selected instrumental variables and harmonization data of blood metabolites on 28-day death in sepsis.**

Notes: IVs: Instrumental Variables, MR Analysis: Mendelian Randomization Analysis.

**Supplementary Figure S1: The LOO analysis of sepsis.**

**Supplementary Figure S2: The LOO analysis of 28-day all-cause mortality in sepsis.**

**Supplementary Figure S3: The scatter plots of sepsis.**

**Supplementary Figure S4: The scatter plots of 28-day all-cause mortality in sepsis.**

**Supplementary Figure S5: The funnel plots of sepsis.**

**Supplementary Figure S6: The funnel plots of 28-day all-cause mortality in sepsis.**
