# Supplementary figures and images for: Investigating the impact of human blood metabolites on the Sepsis development and progression: a study utilizing two-sample Mendelian randomization
Source: Front Med (Lausanne). 2023 Dec 8;10:1310391. doi: 10.3389/fmed.2023.1310391 (PMC10748392; doi:10.3389/fmed.2023.1310391)

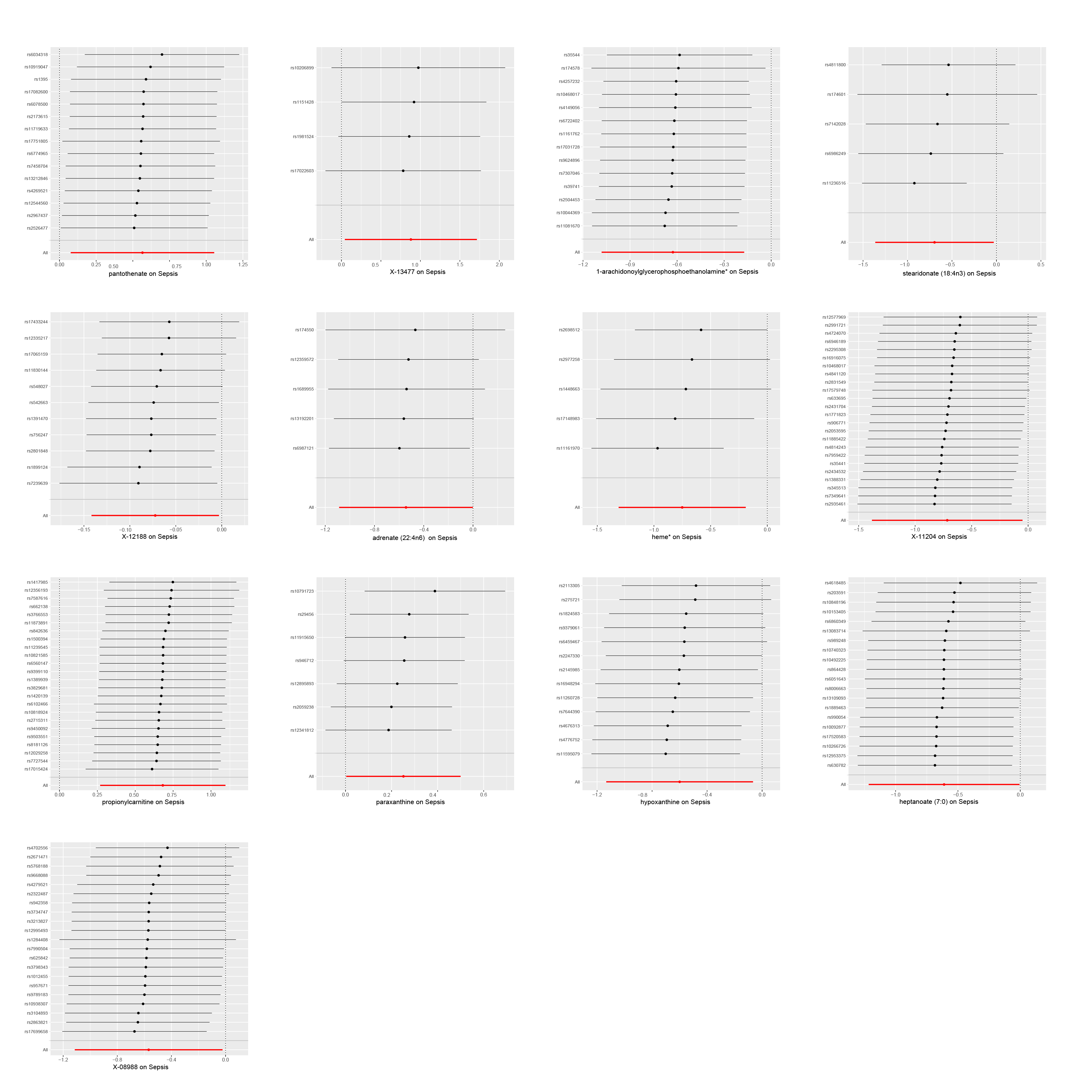

Supplement: Supplementary file 1 [file Data_Sheet_1.zip › Supplementary Figure S1.tif]

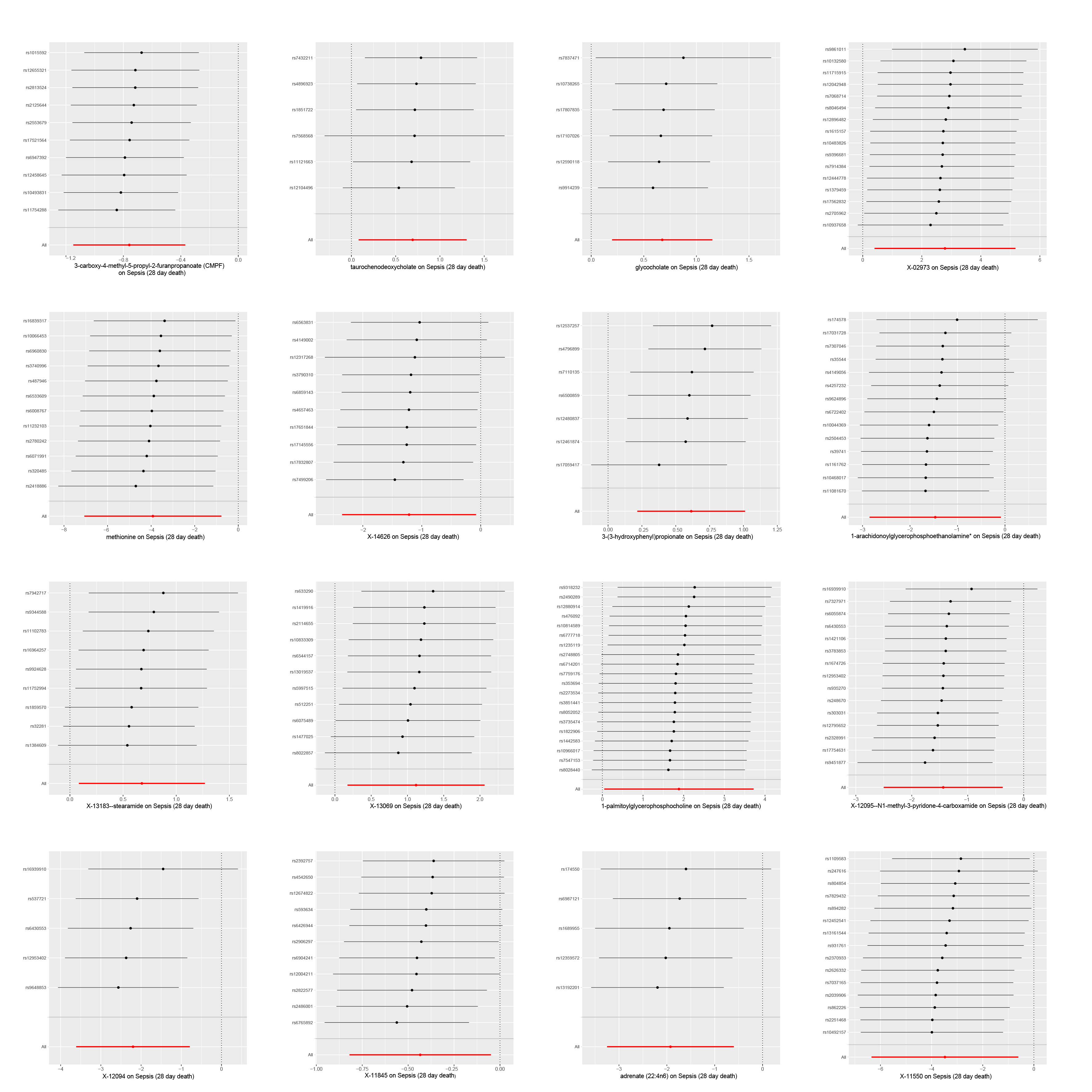

Supplement: Supplementary file 1 [file Data_Sheet_1.zip › Supplementary Figure S2.tif]

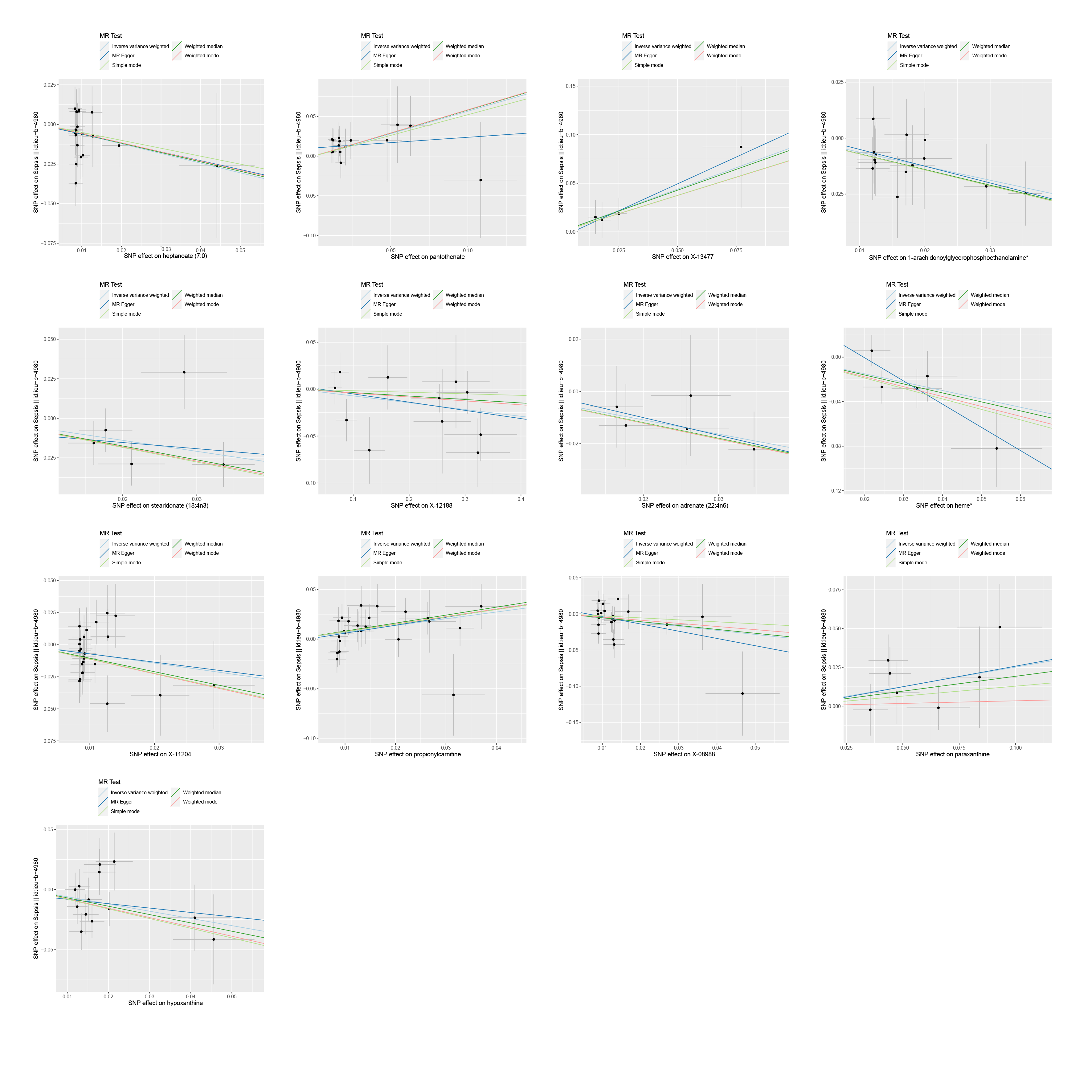

Supplement: Supplementary file 1 [file Data_Sheet_1.zip › Supplementary Figure S3.tif]

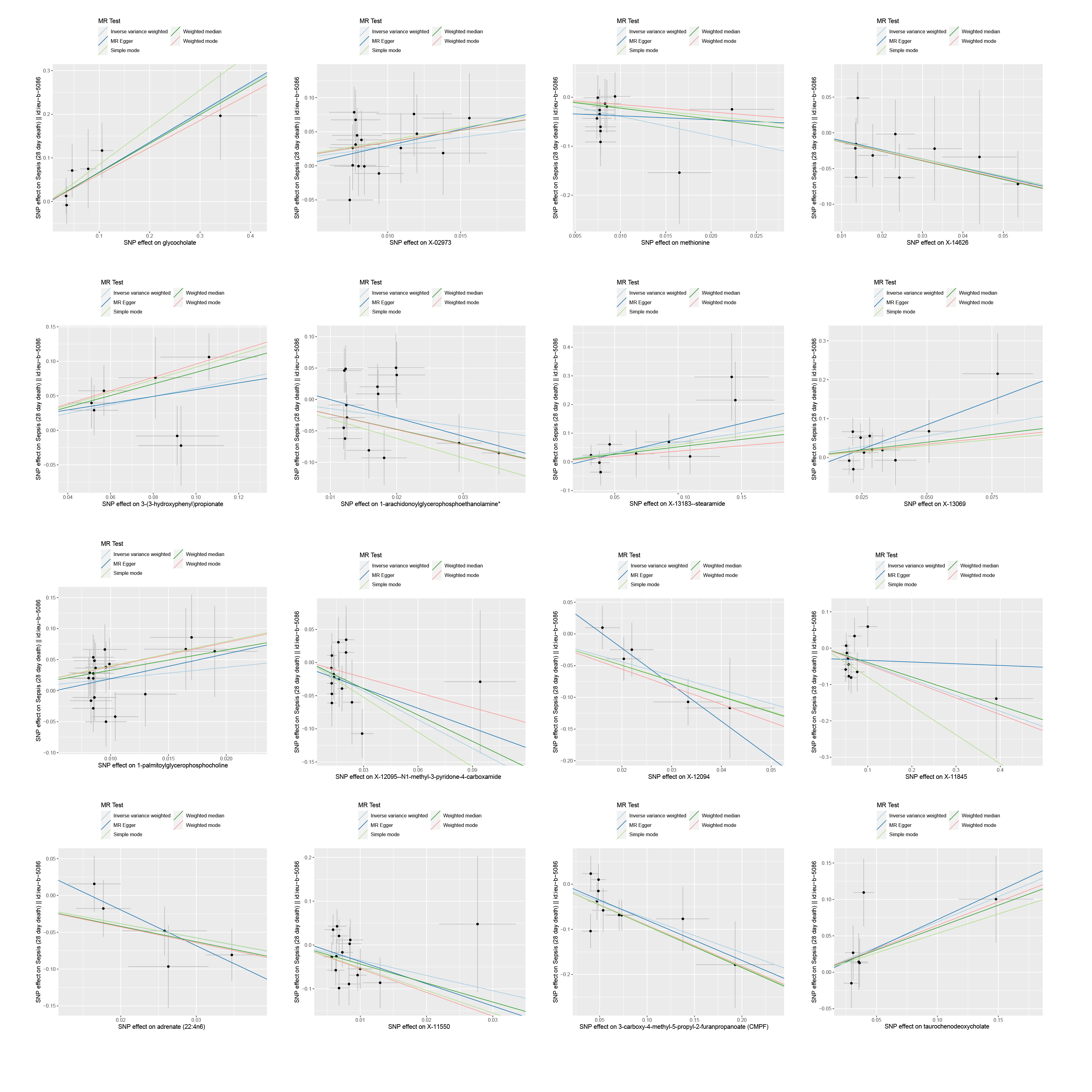

Supplement: Supplementary file 1 [file Data_Sheet_1.zip › Supplementary Figure S4.tif]

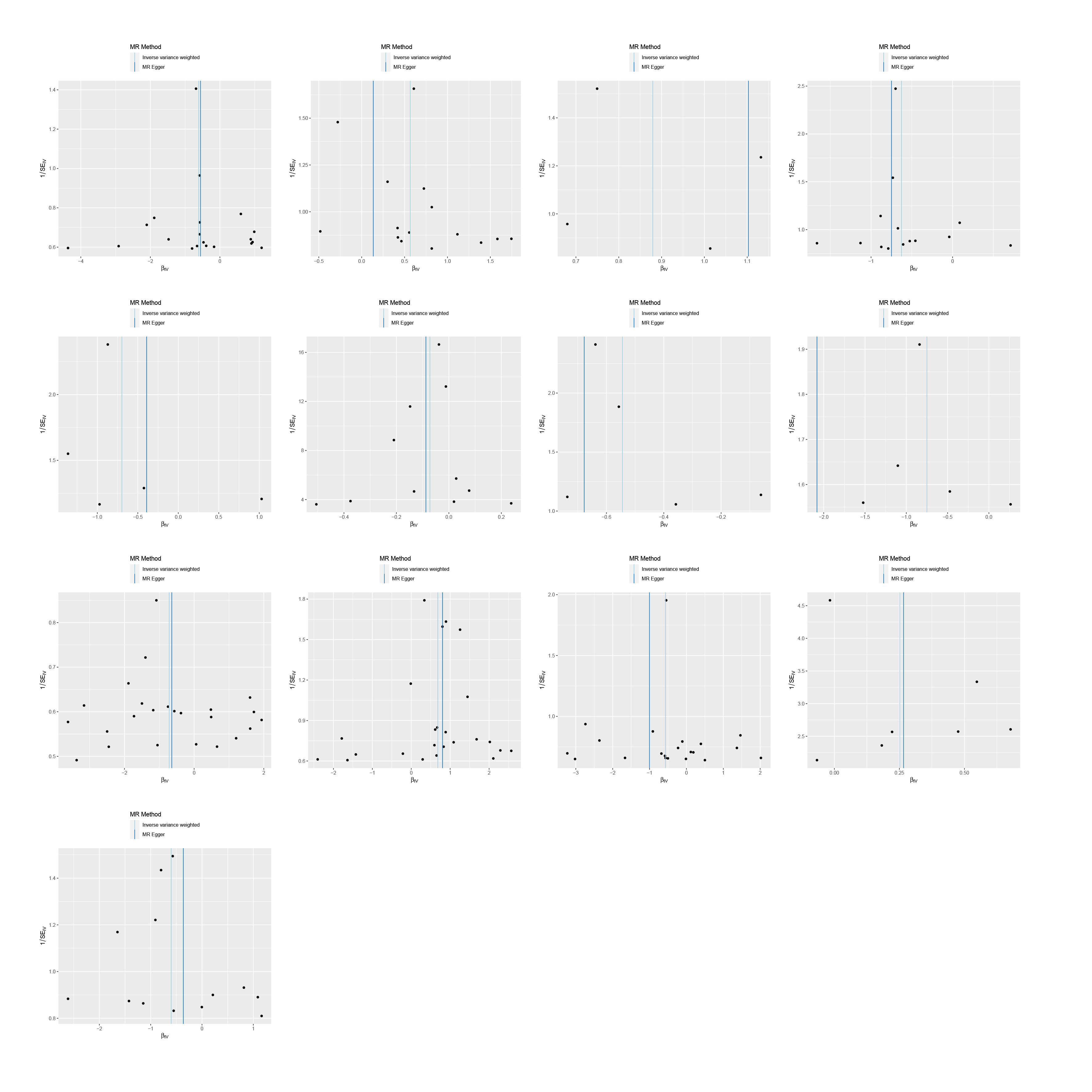

Supplement: Supplementary file 1 [file Data_Sheet_1.zip › Supplementary Figure S5.tif]

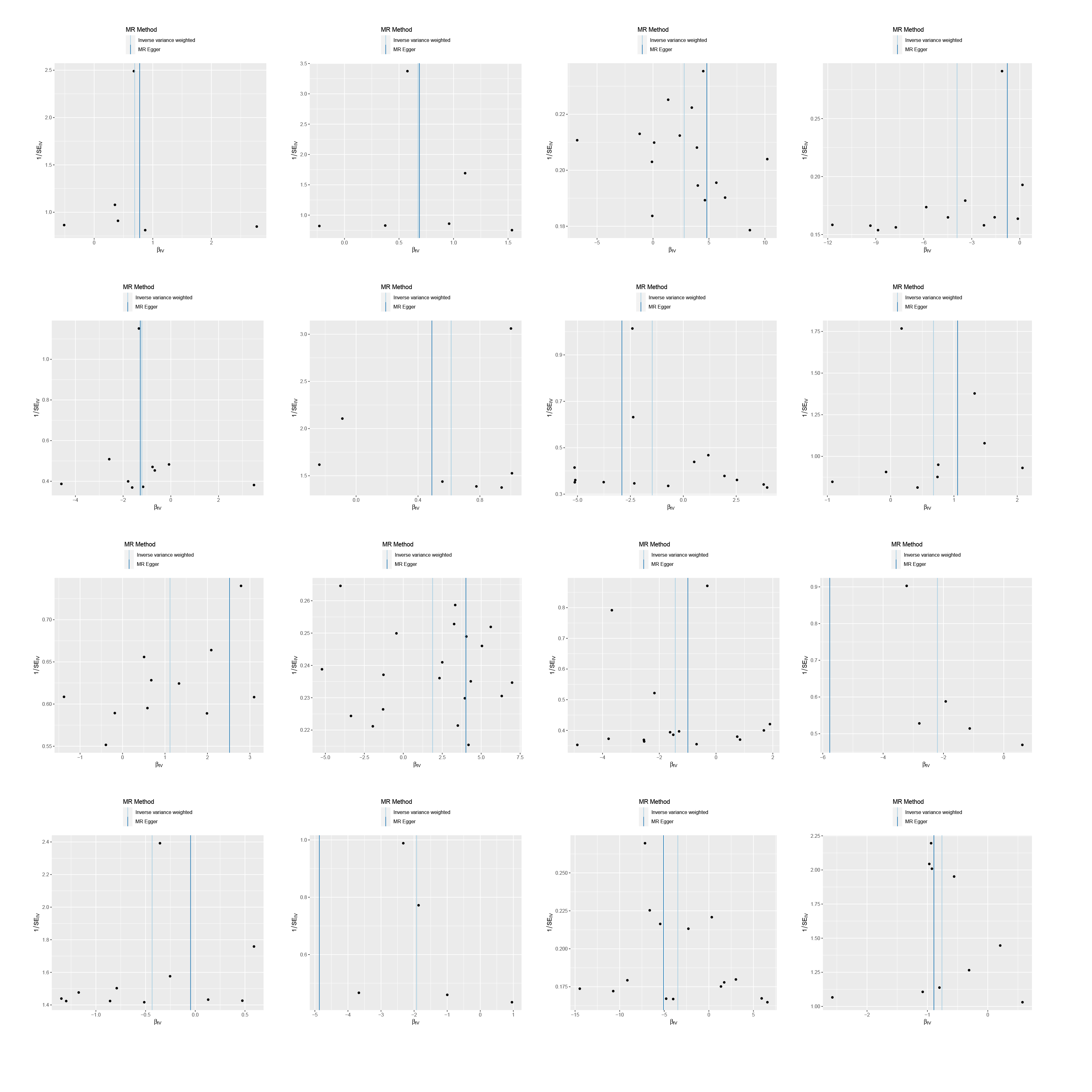

Supplement: Supplementary file 1 [file Data_Sheet_1.zip › Supplementary Figure S6.tif]
